# Vitamin D Impacts on Skeletal Muscle Dysfunction in Patients with COPD Promoting Mitochondrial Health

**DOI:** 10.3390/biomedicines10040898

**Published:** 2022-04-14

**Authors:** Cristina Russo, Maria Stella Valle, Antonino Casabona, Lucia Spicuzza, Gianluca Sambataro, Lucia Malaguarnera

**Affiliations:** 1 Section of Pathology, Department of Biomedical and Biotechnological Sciences, School of Medicine, University of Catania, 95123 Catania, Italy; cristina.russo@unict.it; 2 Section of Physiology, Laboratory of Neuro-Biomechanics, Department of Biomedical and Biotechnological Sciences, School of Medicine, University of Catania, 95123 Catania, Italy; m.valle@unict.it (M.S.V.); casabona@unict.it (A.C.); 3 Department of Clinical and Experimental Medicine, University of Catania, 95123 Catania, Italy; lucia.spicuzza@unict.it (L.S.); dottorsambataro@gmail.com (G.S.)

**Keywords:** vitamin D, COPD, muscle weakness, mitochondria

## Abstract

Skeletal muscle dysfunction is frequently associated with chronic obstructive pulmonary disease (COPD), which is characterized by a permanent airflow limitation, with a worsening respiratory disorder during disease evolution. In COPD, the pathophysiological changes related to the chronic inflammatory state affect oxidant–antioxidant balance, which is one of the main mechanisms accompanying extra-pulmonary comorbidity such as muscle wasting. Muscle impairment is characterized by alterations on muscle fiber architecture, contractile protein integrity, and mitochondrial dysfunction. Exogenous and endogenous sources of reactive oxygen species (ROS) are present in COPD pathology. One of the endogenous sources of ROS is represented by mitochondria. Evidence demonstrated that vitamin D plays a crucial role for the maintenance of skeletal muscle health. Vitamin D deficiency affects oxidative stress and mitochondrial function influencing disease course through an effect on muscle function in COPD patients. This review will focus on vitamin-D-linked mechanisms that could modulate and ameliorate the damage response to free radicals in muscle fibers, evaluating vitamin D supplementation with enough potent effect to contrast mitochondrial impairment, but which avoids potential severe side effects.

## 1. Introduction

Chronic obstructive pulmonary disease (COPD) is a progressive lung disease, characterized by an irreversible airflow limitation [1]. The airway alterations characterizing COPD are obstructive bronchiolitis due to chronic inflammation of peripheral airways and lung parenchyma and emphysema due to the collapse of the alveolar walls and expansion of alveoli. Patients with severe COPD often exhibit overlapping pathologies such as bronchiectasis, lung cancer, hypertension, cardiovascular disease, diabetes mellitus, and osteoporosis [2]. Extra-pulmonary comorbidities also include skeletal muscle dysfunctions. Airflow limitation in patients with COPD decreases the systemic oxygen supply, producing a decline in the aerobic capacity of the type I muscle fibers with a consequent reduction in muscle endurance and a further increase in physical fatigue [3]. Moreover, systemic oxidative stress in patients with severe COPD reduces lower-extremity muscle strength, impairing the functional capacity of locomotor muscles and the ability to perform normal daily activities, such as walking or upright standing. In peripheral skeletal muscles of COPD patients, enzymatic changes and mitochondrial abnormalities have been found [4]. Other muscular abnormalities include a gradual decline of the cross-sectional area, and modifications in the structure of the type of fibers switching from slow-oxidative to fast-glycolytic fiber type [4,5]. As the disease progresses, in many patients, skeletal muscle dysfunction can turn into sarcopenia or cachexia, both of which have been associated with further increased disease severity and mortality risk [6]. Some studies have suggested the importance of optimal vitamin D status on respiratory function, underlining that vitamin D deficiency (VDD) might represent a marker of disease severity [7]. Meta-analysis studies indicate that VDD is directly associated with a more severe disease and with an increased rate of exacerbations and hospitalization [8,9]. Moreover, it has been reported that vitamin D status is linked with muscle strength outcomes [10]. VDD eliciting mitochondrial dysfunction, adenosine triphosphate (ATP) depletion, enhancement of reactive oxygen species (ROS), and oxidative damage leads to muscle atrophy and compromised muscle function [11]. However, several investigations showed that vitamin D supplementation recovers lung function, reduces exacerbations, and improves inspiratory muscle strength and maximal oxygen uptake [12,13]. It also improves physical performance, maximal voluntary ventilation, and inspiratory pressure [14]. In the present review, we examine the molecular mechanisms by which vitamin D has an impact on mitochondria oxidative stress influencing disease course through an effect on muscle function in COPD patients.

## 2. Mechanisms Mediating Muscular Wasting in COPD Patients

In developed countries, most COPD cases are due to cigarette smoking, biomass smoke, and additional environmental pollutants [1]. Carbon monoxide and nicotine damage the endothelium of blood vessels, allowing the adhesion and accumulation of fat in the blood. As a consequence, blood circulation gradually degenerates, and insufficient oxygenation depletes cellular metabolism releasing minor, macro-, and micronutrients to the cells. Altered pulmonary ventilatory mechanics in patients with COPD can further reduce the systemic oxygen supply, producing a decrease in the aerobic cellular metabolism and muscle strength, increasing physical fatigue [15]. Less oxygen distribution to skeletal muscle tissue generates peripheral muscle dysfunction, characterized by reduced muscle strength and subsequent diminished exercise propensity in COPD. Peripheral fatigue leads to progressive muscle disuse with crucial muscle fiber transformations. As muscle activation reduces, the typical tonic activation of the slow motor units, innervating type I fibers, changes into a more intermittent discharge, with significant modification of cellular functions. In fact, a prolonged period of phasic activity applied to oxidative myofibers normally induces oxidative enzymatic reduction, mitochondrial abnormalities, and an increase in myosin heavy chains. As a result, there is a transformation of type I muscle fibers into fatigable type IIx fast-twitch glycolytic fibers [15]. These muscle fiber shifts further increase muscle fatigability, leading to the spiral of progressive inactive lifestyles degenerating into muscle weakness and atrophy [16]. In COPD patients, the impairment in lower limb muscle groups leads to deficits in biomechanical constraints; the balance is altered by anticipatory postural adjustments and motor task transitions [17,18]. Along with adaptations to reduced postural control, they also have slower walking speed with a reduced step length and increased time spent in double support [19]. Muscle dysfunctions in COPD patients are paralleled by the reduction in passive viscoelastic tension produced by the muscular articular system [20]. This finding suggests that disuse may also affect the passive components included in muscles and joints, contributing to the severe motor task alterations associated with COPD linked to muscle dysfunctions [21]. In addition to decreased physical activity, other causes facilitate muscle wasting in COPD including systemic inflammation, oxidative stress, hypoxia, and nutritional depletion [22]. Muscle dysfunction frequently occurs before disease development. This can be considered an index of mortality rate in patients with COPD not negligible compared to lung function [23].

## 3. Vitamin D Metabolism and Biological Function in the Muscle

Vitamin D is a fat-soluble steroid prohormone crucial for calcium homeostasis and bone metabolism [24]. To become active, vitamin D is subjected to two hydroxylation reactions that take place principally in the liver and kidneys [25]. 1α,25(OH)_2_ D3 plasma level is regulated by CYP24A1 (cytochrome P450 family 24 subfamily A member 1) and CYP27B1 (cytochrome P450 family 27 subfamily B member 1). The excess 1α,25(OH)_2_ D3 is regulated by the CYP24A1 enzyme which inactivates the hormone in 24,25-Dihydroxycholecalciferol (1α,24,25(OH)_3_ D3). Conversely, CYP27B1 allows the conversion of 25(OH)D in 1α,25(OH)_2_ D3 [25]. This conversion occurs mostly in the kidney and in all those cells/tissues expressing the CYP27B1 including homeostatic skeletal muscle fibers, in both C2C12 myoblasts and whole mouse muscle [26]. CYP27B1 is an important regulator of the calcium and phosphate homeostatic systems. It is induced by the parathyroid hormone (PTH), low Ca^2+^, and low PO_4_^3−^ levels [27]. 1,25(OH)_2_D synthesis is induced by the parathyroid hormone (PTH), whereas calcium decreasing PTH directs negative feedback from 1,25(OH)_2_D to PTH [28].

The active metabolite 1,25(OH)_2_D modulates the expression of many genes by binding to its nuclear receptor, the vitamin D receptor (VDR) exerting diverse biological effects through genomic and non-genomic activities [29]. Moreover, the vitamin d/VDR complex binding the retinoid X receptor (RXR) forms a VDR-RXR heterodimer which, interacting with genomic vitamin D response elements (VDREs), regulates gene transcription [29]. In humans, the vitamin D system has been shown to be present more in precursor cells than in adult skeletal muscle [30]. Much evidence in the last few decades indicates that vitamin D is involved in skeletal muscle development and regeneration [25]. Moreover, VDR expressed in skeletal muscle induces the synthesis of muscle protein and is required to maintain muscle volume [31]. The addition of 1,25(OH)_2_D to C2C12 myoblasts increases VDR expression, decreases cell proliferation, and promotes myogenic differentiation [32]. The binding of vitamin D to VDR stimulates the intracellular uptake of the inorganic phosphates that are used for producing energy-rich phosphate compounds essential for sustaining muscle contractility [33]. Vitamin D and VDR have an important impact on skeletal muscle function. Subsequently to muscle damage, the moderate expression of VDR and CYP27B1 observed in homeostatic conditions increases significantly. 1,25(OH)_2_D induces the expression of myoblast determination protein 1 (MyoD1) and consequently inhibits myostatin in a time-dependent manner. Furthermore, vitamin D modulates forkhead box O (FOXO) 3 and Notch signaling pathways promoting myoblast self-renewal and sustains the satellite stem cell pool [30].

## 4. Vitamin D Deficiency and Muscle Weakness

Serum 25(OH)D concentration is related to vitamin D exposure and absorption. Therefore, VDD in humans is estimated as serum levels of this metabolite [34]. Serum 25(OH)D < 30 nmol/L (12 ng/mL) is defined as VDD, while 25(OH)D concentrations between 30 and 50 nmol/L (12–20 ng/mL) are categorized as vitamin D insufficiency [29]. At 25(OH)D levels above 30 ng/mL, there are optimum musculoskeletal benefits. A significant association of VDD with muscle dysfunction has been demonstrated [35]. In older adults, plasma 25(OH)D concentrations < 25 nmol/L are associated with significantly lower grip strength [36].

Much evidence suggests that vitamin D supplementation improves muscle strength, although in the different studies conducted up to date, there are different methodological differences such as the characteristics of the participants, the duration, and dosage of supplementation. In a study performed in young adults, which included subjects with a vitamin D status varying from insufficient to optimal, it observed that in those with higher baseline 25(OH)D concentrations, the muscle strength following an intense endurance exercise was recovered [37].

In a meta-analysis study including young participants with 25(OH)D concentrations < 25 nmol/L, vitamin D supplementation ranging from 4000 to 60,000 IU per week strengthened both upper and lower body strength [38].

In a biopsy sample, VDD was found to be linked to skeletal muscle dysfunction and mainly related to type II muscle fiber atrophy [39]. VDR knockout mice (VDRKO) show muscle weakness, muscle fiber atrophy, and hypernuclearity, as well as VDR deletion, generating alterations in muscle function and strength [40,41]. VDRKO exhibited smaller muscle mass and weaker grip strength when compared with controls [42]. Moreover, they showed reduced diameter muscle fibers and an abnormal expression of myogenic transcription factors with respect to wild-type mice, suggesting a physiological role of VDR through temporal down-regulation of myogenic transcription factors [43]. Humans with VDD are often affected by skeletal muscle weakness and myopathy; however, they promptly react to treatment with vitamin D3 [32]. As a consequence of VDD, a pronounced weakness in the proximal muscle becomes evident, which leads to widespread muscle pain and waddling gait [44]. Vitamin D3 supplementation improves muscle strength in humans. Meta-analyses of randomized clinical trials in the general population reported that vitamin D supplementation exerts beneficial effects on muscle strength and physical abilities [45,46]. These improvements are higher in subjects with severe VDD at baseline [47]. Nevertheless, scientific opinions on the relationships between vitamin D status and muscle function in COPD patients are controversial.

## 5. Mitochondria, Oxidative Stress, and Muscular Wasting in COPD Patients

Mitochondria are dynamic organelles playing an important role in cellular energy metabolism. They are the most important source of oxidants within cells and the foremost target of oxidative stress. They form continuously mutable networks within cells. Mitochondrial dynamics are essential to maintain normal cellular processes and are regulated by coordinated fusion and fission cycles. Both mitochondrial structural and functional integrity are influenced by the good organization of mitochondrial quality control, including mitochondrial dynamics and turnover, by preventing mitochondrial network fragmentation and dysfunction, and cell death [48].

**Figure 1:**
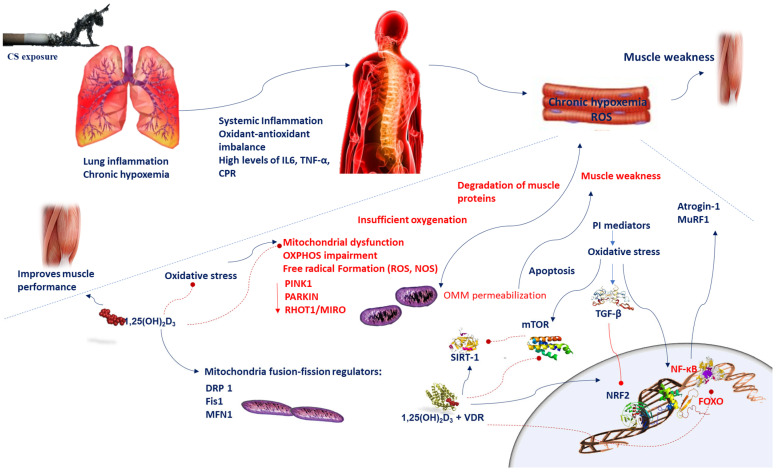
Chronic hypoxemia contributes to inflammation, which generates mitochondrial dysfunction, impairments in mitochondrial turnover, and oxidant–antioxidant imbalance. Reduced respiration causes insufficient oxygenation and mitochondrial dysfunction, which in turn leads to alteration of mitochondrial OXPHOS and increases ROS levels. A disproportionate stimulation of fission induces mitochondrial dysfunction. Reduction in the mitochondrial fusion factor OPA1 impairs the mitochondrial network and promotes apoptosis. Blockade of fission Fis1 or Drp1 inhibits mitochondrial fragmentation. Oxidative stress triggers the TGF-β signaling pathway, which induces inhibitory effect on Nrf2, which in turn inhibits endogenous antioxidants. Oxidative stress induces cellular senescence via FOXO transcription factors and decreases SIRT-1 expression and enzyme activity; ROS activate the PI3K-mTOR pathway. Vitamin D supplementation prevents the mitochondrial dysfunction and oxidative stress by setting MFN1/2, OPA1, and Drp1 expression. Oxidative stress activates NF-κB and FOXO pathways which influences muscle wasting in COPD patients. Vitamin D and VDR represses NF-κB and modulates the post-translational modification and function of FoxO proteins. The beneficial effects of SIRT-1 on mitochondrial function are regulated by vitamin D, which acts by increasing SIRT-1 formation. Abbreviations: CPR = C-reactive protein; Drp1 = dynamin-related protein 1; 1,25(OH)2D3 = 1,25-dihydroxyvitamin D3; Fis1 = fission protein 1; FOXO = forkhead box O; IL-6 = interleukin-6; MFN1/2 = mitofusin-1/2; mTOR = mammalian target of rapamycin; MuRF1 = muscle-specific RING finger protein 1;NOS = nitrogen species; Nrf2 = nuclear factor erythroid 2-related factor 2; NF-κB = nuclear factor kappa; OPA1 = optic atrophy protein 1; OXPHOS = oxidative phosphorylation; PI3K = phosphatidylinositol-3-kinase; ROS = reactive oxygen species; RHOT-1/MIRO = Ras homolog family member T-1; TGF-β = transforming growth factor-beta; TNF-α = tumor necrosis-alpha; sirtuin-1 = SIRT-1; VDR = vitamin D receptor.

Mitochondrial quality control is particularly important to the homeostasis of skeletal myocytes, given their high dependence on oxidative phosphorylation (OXPHOS) for energy supply and their post-mitotic nature, which delays the reduction in mitochondrial dysfunction through cell division. Reduced respiration induces impairment in mitochondrial bioenergetics, which in turn causes the alteration of mitochondrial OXPHOS and reduction in ATP formation and increases ROS production [49]. ROS induce the opening of the mitochondrial membrane permeability transition pore, reduction in mitochondrial b-nicotinamide adenine dinucleotide (NAD^+^) stores, and consequent apoptotic cell injury. Depletion of the mitochondrial fusion factor optic atrophy protein 1 (OPA1) disrupts the mitochondrial network inducing apoptosis [49]. In contrast, the blockade of fission protein 1 (Fis1) or dynamin-related protein 1 (Drp1) prevents mitochondrial fragmentation and apoptosis [50]. Consequently, an unbalanced stimulation of fission can lead to mitochondrial dysfunction (Figure 1).

Inflammation generates mitochondrial dysfunction and oxidative stress and is the main cause that induces the degradation of muscle proteins [51,52]. Mitochondria play an essential role in modulating ROS production [53]. Insufficient oxygenation causes mitochondrial dysfunction, superoxide production, and the intensification of free radical formation influencing membrane potential. The hypoxic stress occurring in COPD patients during exacerbations, or in the presence of chronic respiratory failure, contributes to a low-grade inflammation. In the skeletal muscle tissue of murine models, a low-grade inflammation caused by alveolar hypoxia induces an acute and extensive microvascular inflammation within minutes [54]. Plasma levels of pro-inflammatory mediators such as IL6, TNF-α, and C-reactive protein are persistently elevated and negatively affect muscle mitochondrial function, underscoring the association between low-grade inflammation and sarcopenia [55].

Chronic hypoxemia, generating high levels of ROS in the lungs, is common in patients with COPD and is often associated with muscle oxidative stress [56,57]. Intracellular enzymes generating ROS consist of membrane-bound NADPH oxidases (NOX), a xanthine/xanthine oxidase system, and neutrophil-derived myeloperoxidase (MPO) [57]. Superoxide anions are formed principally by NOX and are moderately weak oxidizing agents; however, they are promptly transformed into more detrimental ROS species, such as the hydroxyl radical and H_2_O_2_, or into potent and very deleterious peroxynitrite radicals formed in the presence of nitric oxide (NO) [58]. Likewise, MPO, produced from activated neutrophils largely present in the airways of patients with COPD, catalyzes the oxidation of chloride, generating very damaging hypochlorous acid. Hypochlorous acid generates by chlorinating tyrosine residues. In the sputum of patients with COPD, 3-chlorotyrosine is remarkably present [59]. Nevertheless, in healthy cells, intracellular antioxidant enzymes efficiently counteract ROS species, reducing their cellular detrimental effects. In the lungs of patients with COPD a plethora of cytokines and chemokines are produced [60].

Several intracellular signaling pathways, including the transcription factor nuclear factor-κB (NF-κB) and signaling molecules, such as Ras/Rac, Jun-N-terminal kinase (JNK), p38 mitogen-activated protein kinase (MAPK), and protein tyrosine phosphatases, induce pro-inflammatory mediators susceptible to oxidative stress.

Particularly oxidative stress activates NF-κB in airway epithelial cells and macrophages of COPD patients [60]. NF-κB activation and the resulting inflammatory effects are involved in muscle wasting of COPD patients [61]. Oxidative stress also induces the transforming growth factor-beta (TGF-β) signaling pathways, which in turn amplifies oxidative stress [62]. The inhibitory effect of TGF-β on nuclear factor erythroid 2-related factor 2 (Nrf2), causing a reduced expression of endogenous antioxidants, may enhance small airway fibrosis [63]. Oxidative stress increases the expression of matrix metallopeptidase 9 (MMP-9), a key enzyme with elastolytic activity involved in the origin of emphysema [52]. MMP-9 enhances elastolysis through oxidative inactivation of α1-antitrypsin and secretory leukoprotease inhibitor, resulting in enhanced neutrophil elastase activity [63]. Oxidative and nitrative stress promote, in exhaled breath condensate and in the sputum of COPD patients, peroxynitrite formation, which increases protein tyrosine nitration, that in turn impairs protein structure and function and further damage of endothelial function in COPD patients [53]. Additionally, through NO signaling, inflammation may influence muscle mitochondrial function. NO influences mitochondria functions by controlling biogenesis, O_2_ depletion, and redox homeostasis [64]. In skeletal myofibers, NO competition with O_2_ could optimize O_2_ repartition between subsarcolemmal mitochondria and interfibrillar mitochondria [65]. Alternatively, the increase in NO formation by inducible NOS (iNOS) results in a significant electron transport chain inhibition, amplification of oxidant production, and induction of apoptosis via outer mitochondrial membrane (OMM) permeabilization [66]. In mice, chronic pulmonary inflammation due to permanent TNFα overexpression strongly impairs skeletal muscle tissue [67]. Mitochondrial turnover deterioration is another mechanism connecting inflammation to mitochondrial dysfunction [68]. TNF-α strongly activates iNOS and affects NO signaling, which provides a mechanistic connection between inflammation and mitochondrial dysfunction. Moreover, TNF-α is an inducer of apoptosis through the death-receptor signaling pathway [69,70]. Downstream of TNF-α, caspase-8 starts the caspase cascade and acts as a bridge between the extrinsic and intrinsic apoptotic pathways via proapoptotic protein (Bid) truncation [71,72]. Then, Bid promotes OMM permeabilization delivering apoptogenic factors from the intermembrane space [72]. Moreover, elevated levels of TNF-α decrease the mRNA abundance of peroxisome proliferator-activated receptor gamma coactivator 1α (PGC1α), transcription factor A mitochondrial (TFAM), and nuclear respiratory factor 1 in cultured C2C12 myoblasts, indicating the suppression of mitochondriogenesis [73] (Figure 1). The chronic pulmonary inflammatory response has an important impact on muscle oxidative capacity, via citrate synthase activity and PGC1α mRNA expression and decreased oxidative IIa muscle fibers [67]. Therefore, the augmented oxidative stress in skeletal muscle causes a shift towards a-type IIx-oriented muscle phenotype with a reduced ability to distribute and use oxygen [74]. Systemic inflammation and oxidant–antioxidant imbalance could induce modifications in cellular phenotypes all over the organism, impairing organ and tissue homeostasis. In addition, inflammation by preventing autophagy could intensify mitochondrial dysfunction [75]. Oxidative stress can induce cellular senescence via FOXO transcription factors and then reduce sirtuin-1 enzyme activity [76] and expression, which is associated with increased expression of MMP-9 and acetylation of NF-κB [76]. Moreover, ROS trigger the PI3K-mTOR (mammalian target of rapamycin) pathway promoting an increased microRNA-34a, which inhibits sirtuin-1 (SIRT-1) [77] (Figure 1). Therefore, targeted interventions on inflammation should prevent muscle deterioration and function.

## 6. Mitochondrial Alterations and Muscular Wasting in COPD Patients

Mitochondrial quality control disorders causing mitochondrial dysfunctions contribute to muscle loss associated with atrophying conditions [78]. Mitochondrial dysfunction includes increased mitochondrial reactive oxygen species (mtROS) generation and reduction in membrane potential, OXPHOS, and crucial mitochondrial proteins such as PTEN-induced putative kinase 1 (PINK1), PARKIN, RHOT1/MIRO, and DRP [79]. Basically, fusion allows joining mitochondria to merge their contents, thus reorganizing proteins, mitochondrial DNA (mtDNA), and metabolites and equilibrating the concentrations of nuclear-encoded proteins across organelles [80]. Imbalances in fusion–fission have been proposed as a formation mechanism of aberrant mitochondria under stress conditions. Fission segregates components of the network that are permanently damaged or unnecessary, for subsequent removal [48]. Unbalance in either of these processes generates altered cellular physiology and mitochondrial function [80].

Therefore, it is possible that muscle deterioration in COPD patients is due to a fusion among mitochondria. mtDNA is susceptible to oxidative stress, attributable to its contiguity to the source of oxidants and the delicate repair system compared with nuclear DNA and the absence of histones and introns [81]. Mitochondrial dysfunctions arising from oxidative damage to mtDNA can produce a vicious cycle in which the synthesis of defective electron transport chain (ETC) subunits results in OXPHOS impairment, decreased ATP production, and additional ROS generation [82]. A rapid muscle impairment generates protein breakdown and the deterioration of the anti-oxidative pathways; consequently, mitochondrial damage is also induced in the course of exacerbations [83]. Therefore, an integration of the total cellular mtDNA pool takes place, disturbing the association between genotype (i.e., damaged mtDNA) and phenotype (i.e., increased ROS production and/or malfunctioning OXPHOS) [81]. The exposure of cultured cells to sub-cytotoxic doses of hydrogen peroxide (H_2_O_2_) represses the expression of fission protein 1 (Fis1), thus promoting the formation of elongated mitochondria with augmented oxidant emission [84] (Figure 1). Upregulation of fusion and/or downregulation of fission could serve to maintain the viability of myocytes until mtDNA suffers damage and consequently mitochondrial interconnection prevents the elimination of impaired mitochondria [81].

The expression of the fission machinery in a transgenic mouse model induced mitochondrial dysfunction, remodeling of the mitochondrial network, protein breakdown, and fiber atrophy [85]. Mitochondrial dysfunctions are frequently observed in COPD and are important events arising in the pathogenesis of this disease [86]. Acute cigarette smoke (CS) exposure increases the levels of DRP1 and FIS1; hence, increasing mitochondrial fragmentation [87] decreases the outer mitochondrial membrane (OMM) fusion proteins such as MFN1 [86] (Figure 1). In addition, a permanent low dose-rate of CS exposure in primary bronchial epithelial cells (PBECs) of COPD patients disrupts mitophagy and induces the augmentation of damaged mitochondria and a rise in the fusion proteins mitofusin 1/2 (MFN1/2) and OPA1, resulting in merged mitochondria in PBECs [86,88].

## 7. Role of Vitamin D in Anti-Oxidative Mechanisms Implicated in Muscular Wasting in COPD Patients

Vitamin D regulates calcium (Ca^2+^) homeostasis in skeletal muscle. Ca^2+^ is an important component in muscle energy metabolism contributing to the interaction between cytosol and mitochondria [89]. Ca^2+^ and ROS are the main secondary messengers involved in numerous cellular signaling pathways. Mitochondria influence both ROS and Ca^2+^ homeostasis and transfer signaling [90]. The compromised Ca^2+^ protecting role of dysfunctional mitochondria generates an increase in the intracellular level of Ca^2+^. Thus, VDD may cause mitochondria’s insufficient Ca^2+^ uptake generating the perturbations of cellular metabolic homeostasis [11]. 1α,25(OH)_2_D_3_, the most active form of vitamin D3, is crucial in muscle regeneration, in the regulation of skeletal muscle tone and contraction, as well as in the preservation against muscle damage [11]. VDD modifies muscle contraction kinetics reducing Ca^2+^ reuptake into the sarcoplasmic reticulum, thus leading to a perpetuation of the relaxation phase of muscle contraction. Moreover, VDD increases the cytotoxicity mediated by ROS and is associated with failure in mitochondrial respiration [88]. Therefore, VDD may contribute to exacerbating the damage of muscle and atrophy for the excess mitochondrial ROS production [11], oxidative impairment, and ATP reduction. In several clinical models, muscle atrophy and deficits in muscle strength at low 25(OH)D concentrations (<50 nmol/L) levels have been reported [91]. In muscle, one of the wasting reasons results from a disproportion in the protein degradation and synthesis rates [19]. VDD influences protein synthesis and degradation. The foremost known proteolytic pathways in the skeletal muscle are the ATP-ubiquitin-dependent system, the lysosomal system, and the cytosolic calcium-activated system [92]. The ATP-ubiquitin-dependent system is the only one dependent on vitamin D [92]. In skeletal muscle cells, the treatment with 1α,25(OH)_2_D_3_ induces an increment of oxygen consumption rate (OCR) and the generation of ATP [45]. In addition, vitamin D status mediates changes in skeletal muscle mitochondrial dynamics, pyruvate dehydrogenase phosphorylation, and expression of nuclear genes encoding mitochondrial proteins, and influences skeletal muscle performance [93]. Nevertheless, direct treatment of isolated mitochondria with 1α,25(OH)_2_D_3_ fails to increase OCR, showing that the effects of 1α,25(OH)_2_D_3_ on OCR might be VDR-dependent or by other extra-mitochondrial biochemical events (Figure 1) [91]. Therefore, vitamin D is beneficial in the treatment of muscle weakness once metabolized to 1α,25(OH)_2_ D_3_ [45]. This process may occur when VDD and high PTH levels drive the rapid metabolism of 25(OH)D_3_ to 1α,25(OH)_2_D_3_. A treatment with cholecalciferol in VDD humans increases the mitochondrial oxidative phosphorylation rate [91]. Furthermore, VDD affects alterations in antioxidant enzyme activities [11]. In skeletal muscle, VDD also impacts on nitrosative stress, lipid and protein peroxidation, and reduced activities of the antioxidant enzymes [11,91]. C2C12 cell lines treated with 1,25(OH)D showed a decrease in ROS synthesis, lipid and protein oxidation, protein ubiquitination, intracellular damage, muscle proteolysis, and atrophy markers, and an increase in glutathione peroxidase (GPx) and superoxide dismutase (SOD) activities and markers of mitochondrial biogenesis in paraspinal muscle [92]. Optimal levels of ROS are used for signal transduction by skeletal muscle cells following damage. While exaggerated production of ROS by disabling defensive antioxidant systems, it damages muscle integrity [91]. Treatments of hyperoxia-exposed animals with 1,25(OH)2D3 cause a significant increase in body weight and reduced hyperoxia-induced lung injury [94]. Experimental studies on mice show that 12 months’ VDD decreases anaerobic capacity and lean mass, and promotes a trend towards smaller fast-twitch fiber cross-sectional area and gait disturbance, resulting in sarcopenia. Additionally, VDD mice displayed an increase in atrophy-associated atrogin-1 gene expression and differential expression of muscle-regulation-associated miR-26a compared to control mice [95]. This finding strongly confirms that VDD impacts muscular deterioration. Indeed, vitamin-D-treated rats displayed a reduction in both oxidative stress and tissue damage after full exercise [95]. These data corroborate the notion that vitamin D controls mitochondrial function and oxidative stress in skeletal muscle. In several experimental models, vitamin D analogues exert a protective effect on skeletal muscle and cells experiencing oxidative stress [96]. The explanation of the mechanism by which vitamin D regulates oxidative stress may be dependent on its influence in the regulation of mitochondrial dynamics and function. Pulmonary VDR and Nrf-2 are reduced in COPD patients [97] (Figure 1). Nrf-2 is an important transcription factor that mediates antioxidant defense pathways [98]. Pulmonary Nrf-2 downregulation causes failure of the antioxidant defense system, impairment of pulmonary epithelial cells, and promotes the onset of COPD [97]. Vitamin D supplementation activates VDR [99] and induces the Nrf2-Keap1 antioxidant pathway [98] (Table 1).

These data highlight the value of vitamin D for a suitable redox equilibrium and underline how vitamin D analogues’ administration stimulates muscle mitochondrial health during oxidative stress.

## 8. Mitophagy Alterations and Muscular Wasting in COPD Patients

As mentioned above, systemic inflammation by reducing mitochondrial biogenesis and promoting mitophagy and autophagy in myocytes adversely affects mitochondrial function [86]. Consequently, the inflammatory process generates skeletal muscle dysfunction in COPD patients.

Dysfunctional or impaired mitochondria are removed from the cell via mitophagy, whereby altered mitochondria are incorporated into the autophagosomes and subjected to lysosomal degradation and selective autophagy [103].

Defective mitochondria increase cellular oxidative stress and induce cell death [88]. Mitochondrial dynamics and apoptotic signaling are closely correlated. This relationship is essential to regulate cell death/survival pathways.

There are two forms of mitophagy: receptor-mediated mitophagy and ubiquitin-mediated mitophagy [103]. The mitophagy receptors participating in the selective clearance of mitochondria include NIX, BNIP3 (BCL2 and adenovirus E1B 19-kDa-interacting protein 3), also referred as NIX-BNIP3-FUDNC1, as well as PINK1/Parkin pathway [104].

**Figure 2:**
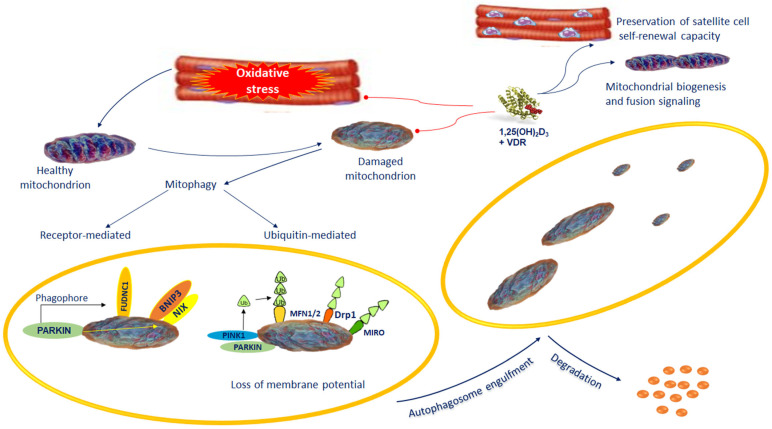
Dysfunctional or impaired mitochondria are removed from the cell via mitophagy. Defective mitochondria are loaded into the autophagosomes followed by lysosomal degradation. Mitophagy takes place by selective autophagy of mitochondria via receptor-mediated mitophagy or ubiquitin-mediated mitophagy. The mitophagy receptors participating in the selective clearance of mitochondria include BNIP3/NIX and FUDNC1 and the PINK1/Parkin pathway. The preservation of the normal mitochondrial control is one of the most important actions of vitamin D. During muscle regeneration, 1,25(OH)2D3 stimulates an increase in VDR levels in satellite cells and central myonuclei. This process subsidizes the preservation of satellite cell self-renewal capacity. VDR increases mitochondrial biogenesis and fusion signaling, and inhibiting ROS production mitigates antioxidant demand, which promotes regenerative phenotype. Abbreviations: BNIP3/NIX: BCL2 interacting-protein-3-like; FUNDC1 = FUN14 domain-containing 1; 1,25(OH)_2_D3 = 1,25-dihydroxyvitamin D3; MFN 1/2 = Mitofusin 1/2; Parkin = Parkinson protein 2, E3 ubiquitin protein ligase; PINK1 = PTEN-induced putative kinase protein-1; RHOT1/MIRO: Ras homolog family member T; VDR = Vitamin D receptor.

Mitochondrial membrane depolarization initiates mitophagy followed by PINK1 stability, which controls mitochondrial quality via fission on mitochondrial outer membranes. PINK1 induces Parkin, an E3 ubiquitin ligase. Parkin cooperates with PINK1 in mitochondrial quality control marking damaged mitochondria [105] (Figure 2). Once recruited to mitochondria, Parkin ubiquitinates MFN2. MFN2 acting as a receptor for Parkin is essential for its localization of damaged mitochondria and the subsequent interaction with membranes containing protein microtubule-associated protein light chain 3 (LC3) to sequestrate mitochondria and the formation of autophagosomes (Figure 2).

The pathways involved in mitophagy regulation ultimately cause the recruitment of proteins regulating autophagy to promote autophagosome formation [106]. The expression levels of the autophagy-related genes LC-3B, BNIP3, and GABARAPL1 are increased in skeletal muscle during pulmonary inflammation; however, they display a differential dependence on muscle NF-κB [106]. NF-κB-independent elevation of BNIP3 transcripts may be the consequence of FOXO3 of rapid atrophy [107]. FOXO3 activation causes the transcription of the atrogin-1 promoter and an intense decrease in myotubes and hence fiber size (Figure 1) [108]. Clinically, this process could lead to a gradual decline in muscular performance and the evolution of muscle atrophy.

## 9. Role of Vitamin D in Mitochondrial Biogenesis and Dysfunction in Muscular Wasting of COPD Patients

In cellular bioenergetics, the preservation of the normal mitochondrial control is one of the most important actions of vitamin D [100]. VDD inhibits VDR signaling, promotes oxidative stress, and reduces mitochondrial biogenesis and function.

Therefore, the deleterious effects of permanent VDD on the mitochondrial function fatally leads to muscle atrophy.

During muscle regeneration, 1,25(OH)2D3 induces VDR in satellite cells and central myonuclei promoting their proliferation cell self-renewal capacity and differentiation (Figure 2). In addition, VDR activation by preventing oxidative stress promotes mitochondrial biogenesis and fusion, and by attenuating antioxidant consumption, facilitates the regenerative process. Vitamin D supplementation reduces the oxidative stress and mitochondrial dysfunction and improves the mitochondrial cristae structure by regulating the expression of MFN1/2, OPA1, and Drp1.

So far, one of the best pathways recognized of mitophagy in mammals is that of PINK1/Parkin [106] (Figure 1).

Vitamin D supplementation in symptomatic VDD subjects improves mitochondrial oxidative function and skeletal muscle function and performance (Table 1) [100].

A prominent role in skeletal muscle mitochondrial biogenesis is carried out by PGC transcription factors. Interestingly, high PGC-1α levels also avoid transcriptional activity of FOXO3a. This observation indicates that mitochondria might participate in the atrophy progression [109]. In fact, FOXO factors control several atrophy-related genes recognized as “atrophy patterns” present in various atrophy types and stimulate the expression of many and enhance protein degradation [110]. Moreover, FOXOs impede cell cycle progression and activate apoptosis [111].

VDD reduces PGC-1α and IGF-1 via VDR—the nuclear receptor. It has been reported that VDR signaling improved by vitamin D stimulation inhibited FOXO1 expression, nuclear translocation, and activity in C2C12 muscle cells. The inhibition of FOXO1 activation vanished if VDR was suppressed. Therefore, FOXO1 is a strategic regulator of VDR signaling in skeletal muscle atrophy [101].

Akt is involved in the progression of muscle atrophy [112]. FOXO3 action is inhibited by Akt, which, by phosphorylation of FOXO3-conserved residues, causes their inactivation compromising their action towards target genes [112]. FOXO3a phosphorylation prevents its translocation to the nuclei, and consequently the expression of target genes for muscle atrophy, including F-box protein (MAFbx) and MuRF, are inhibited. The signaling pathway inducing the expression of MuRF1 and MAFbx includes Src-ERK1/2-Akt-FOXO [113]. In addition, Akt regulates muscle synthesis via mTOR. In murine C2C12 skeletal myotubes, 1α,25(OH)_2_D_3_ stimulates the Akt/mTOR-dependent pathway, inducing activation of their protein synthesis [109] (Figure 1).

Remarkably, SIRT-1 modulates VDR signaling. Further, sirtuin-1 controls inflammation, oxidative stress, mitochondrial function and biogenesis, apoptosis, and cellular senescence. SIRT1-catalyzed deacetylation promotes FOXO activity and DNA binding affinity [114].

Vitamin D inducing the formation of SIRT-1 [102] exerts positive effects on Sirt1 and mitochondrial function (Table 1). Moreover, VDR controls the function of FOXO proteins and acts as a selective regulator of SIRT-1 action [114] (Figure 1).

It has been reported that the abolition of VDD recovers fatigue and myopathy signs in VDD subjects [100]. Other investigators found that in patients with low back pain, vitamin D supplementation reduced oxidative stress in skeletal muscle [115]. Nevertheless, it was demonstrated that the C2C12 cell line and primary myoblasts’ vitamin D over dosage causes VDR overexpression and damages their differentiation into mature myotubes [101]. Some other studies indicate that moderate over dosage of vitamin D causes adverse effects on skeletal muscle function [110,116]. Likewise, the supraphysiological dose of 1α,25(OH)_2_D_3_ injected into experimentally injured muscle disturbs the regenerative response in muscle, reduces satellite cell differentiation, interrupts regenerative muscle fiber formation, and induces muscular fibrosis [31].

## 10. Concluding Remarks and Future Prospective

**Figure 3:**
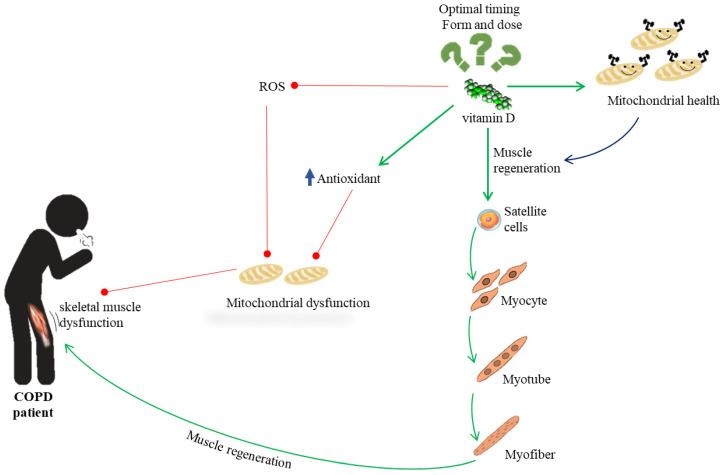
Overview of the possible role of vitamin D activity in patients with COPD suffering from skeletal muscle dysfunction.

Much evidence suggests a possible role of vitamin D activity in patients with COPD suffering from skeletal muscle dysfunction. In vitro and in vivo studies on animal models have shown that vitamin D mitigates the production of ROS, increases antioxidant capacity, and prevents oxidative stress, which is an important component in muscle damage. In addition, suppression of VDR causes a decrease in mitochondrial oxidative capacity and ATP production, indicating that vitamin D is crucial for mitochondrial oxidative phosphorylation capability, which is important for muscle regeneration. Muscle regeneration is an intricate process that includes repair of mitochondrial function and activation of the resident skeletal muscle stem cells, named satellite cells. VDR expression is significantly upregulated following injury, mainly in central nuclei and satellite cells subsequent to muscle injury [42]. Thus, the involvement of vitamin D in the mitochondrial integrity may also include satellite cell activity and self-renewal aptitude, which affect muscle recovery. Nevertheless, the exact mechanism used by vitamin D to promote muscle health in COPD patients currently has not been fully clarified (Figure 3), nor the optimal form, dose, and time of administration.

Further research is necessary to define the mechanistic action of vitamin D on mitochondria, and the modalities by which these processes take place in the muscular dysfunction of subjects with COPD. In addition, levels of vitamin D sufficiency reduction should be standardized, as well the duration of vitamin D administration, and there should be a comparison of several analogues of vitamin D to clarify its potential as an important supplement for the recovery of musculoskeletal function in COPD patients.

## Figures and Tables

**Table 1:** Possible vitamin D linked mechanisms involved in mitochondrial function.

Experimental Data	Model	Sample Size	Tissue	Approach	Reference
Evaluation of oxygen consumption, biogenesis, dynamics, and nuclear genes encoding variations.	Human	//	Muscle biopsies. Primary human skeletal muscle cells.	Supplementation of 1α,25-Dihydroxyvitamin D3 (10-8 M) for 48 h. VDR expression in human muscle cells and skeletal muscle homogenates. Effect of 1α,25(OH)_2_D mitochondrial oxygen consumption and in expression of: mitochondrial proteins that alter mitochondrial fusion; proteins associated with mitochondrial fission; phosphorylated pyruvate dehydrogenase and pyruvate dehydrogenase kinase 4; genes encoding mitochondrial proteins; and genes encoding cellular signaling and growth-regulatory pathways in adult human skeletal muscle cells. Knockdown of VDR with silencing RNA in skeletal muscle cells to detect the effects of 1α,25(OH)_2_D_3_ on OCR.	[45]
Assessment of oxidative and nitrosative stress parameters.	Rat	//	Fasting blood samples analysis. C2C12 cell culture.	Supplementation of 1,25(OH)2D3 (1 nM and 10 nM) for 24 h. Effect of VDD in muscle oxidative stress in a rat model. Pre/post treatment of C2C12 muscle cells with vitamin D offers protection against oxidative stress induced muscle proteolysis. VDD increase in activities of the glutathione-dependent enzymes and decrease in SOD and catalase enzymes in the rat muscle. Pre/post treatment of C2C12 muscle cells with vitamin D correct total protein degradation, 20S proteasomal enzyme activity, muscle atrophy gene markers and expression of proteasome subunit genes induced by oxidative stress.	[91]
Serum and lung tissue analysis.	Human	180 COPD patients	Human lung tissues and serum samples of COPD.	Level of pulmonary VDR-positive nuclei between COPD patients and control subjects Correlations of pulmonary function with pulmonary DJ-1, Nrf-2 and VDR in COPD patients.	[97]
Antioxidant and antiaging effects of 1,25Dihydroxyvitamin D by activation of Nrf2-antioxidant signaling and inactivation of p16/p53-senescence signaling.	Mouse	120	Skin, lung, liver, kidney, and spleen.	Two different supplementation: thrice weekly of 2.2 IU vitamin D/g or 1,25(OH)2D3 (1 μg/kg) until death. Effects of a high-calcium/phosphate diet, of 1,25(OH)_2_D_3,_ and of antioxidant supplementation on lifespan, body weight, skin morphology; on oxidative stress, DNA damage, protein expression of oncogenes and tumor suppressive genes; and on cell proliferation and senescence in 1α(OH)ase^−/−^ mice.	[98]
Improvement in parameters of mitochondrial function in vitamin-D-deficient individuals after vitamin D supplementation.	Human	12 subjects with severe vitamin D deficiency	Serum samples	Effect of cholecalciferol therapy (20 000 IU supplementation on alternate days for 10–12 weeks) in muscle mitochondrial maximal oxidative phosphorylation after exercise in symptomatic, vitamin-D-deficient individuals.	[100]
FOXO1 activation in the skeletal muscle of global VDR-null mice.	Mouse		VDR^−/−^ mice administered a diet enriched with calcium and phosphorus; SMVDR^−/−^ mice generated by crossing VDR^loxp/loxp^ mice with mice with muscle-specific Cre recombinase expression under the control of the myosin light chain 1f (MLC 1f) genomic locus; C2C12 muscle cells.	Treatment of C2C12 muscle cells with 1,25-dihydroxyvitamin D (100 nM for 48 h) to detect FOXO1 expression, nuclear translocation, and activity. Evaluation of FOXO1 activation in knockdown VDR mice.	[101]
Effect of vitamin D supplementation on oxidative stress.	Mouse	Eight mice for each experimental group.	Adipocyte cell culture model	Supplementation of cholecalciferol (67 IU VD/kg daily for last 8 weeks) to detect the effects of 1,25(OH)_2_D_3_ supplementation in NOX4, Nrf2 SIRT-1 expression, ROS production, NF-κB and AMPK phosphorylation.	[102]

Abbreviations: VDR = vitamin D receptor; 1α,25(OH)_2_D = 1α,25-Dihydroxyvitamin D; RNA = ribonucleic acid; 1,25(OH)2D3 = 1,25-dihydroxyvitamin D3; OCR = oxygen consumption rate; VDD = vitamin D deficiency; SOD = Superoxide dismutase; Nrf2 = nuclear factor erythroid 2-related factor 2; 1α(OH)ase = 1α-hydroxylase enzyme; FOXO1 = forkhead box O1; SMVDR = skeletal muscle-specific VDR; NOX4 = NADPH Oxidase 4; SIRT-1 = sirtuin-1; ROS = reactive oxygen species; NF-κB = nuclear factor kappa; AMPK = AMP-activated protein kinase.
